# Automatic classification of canine thoracic radiographs using deep learning

**DOI:** 10.1038/s41598-021-83515-3

**Published:** 2021-02-17

**Authors:** Tommaso Banzato, Marek Wodzinski, Silvia Burti, Valentina Longhin Osti, Valentina Rossoni, Manfredo Atzori, Alessandro Zotti

**Affiliations:** 1 Department of Animal Medicine, Productions, and Health, Legnaro (PD), University of Padua, 35020 Padua, Italy; 2 Department of Measurement and Electronics, AGH University of Science and Technology, 32059 Kraków, Poland; 3 Information Systems Institute, University of Applied Sciences Western Switzerland (HES-SO Valais), 3960 Sierre, Switzerland; 4 Department of Neuroscience, University of Padua, 35128 Padua, IT Italy

**Keywords:** Medical imaging, Radiography

## Abstract

The interpretation of thoracic radiographs is a challenging and error-prone task for veterinarians. Despite recent advancements in machine learning and computer vision, the development of computer-aided diagnostic systems for radiographs remains a challenging and unsolved problem, particularly in the context of veterinary medicine. In this study, a novel method, based on multi-label deep convolutional neural network (CNN), for the classification of thoracic radiographs in dogs was developed. All the thoracic radiographs of dogs performed between 2010 and 2020 in the institution were retrospectively collected. Radiographs were taken with two different radiograph acquisition systems and were divided into two data sets accordingly. One data set (Data Set 1) was used for training and testing and another data set (Data Set 2) was used to test the generalization ability of the CNNs. Radiographic findings used as non mutually exclusive labels to train the CNNs were: unremarkable, cardiomegaly, alveolar pattern, bronchial pattern, interstitial pattern, mass, pleural effusion, pneumothorax, and megaesophagus. Two different CNNs, based on ResNet-50 and DenseNet-121 architectures respectively, were developed and tested. The CNN based on ResNet-50 had an Area Under the Receive-Operator Curve (AUC) above 0.8 for all the included radiographic findings except for bronchial and interstitial patterns both on Data Set 1 and Data Set 2. The CNN based on DenseNet-121 had a lower overall performance. Statistically significant differences in the generalization ability between the two CNNs were evident, with the CNN based on ResNet-50 showing better performance for alveolar pattern, interstitial pattern, megaesophagus, and pneumothorax.

## Introduction

Thoracic radiographs are part of routine clinical evaluation of patients with confirmed or suspected thoracic pathology both in human and veterinary medicine. Nevertheless, interpreting thoracic radiographs is a challenging and error-prone task for the medical doctor^1,2^, and for the veterinary practitioner alike^3^. In human medicine, despite the efforts to improve radiology residents’ training programmes, the prevalence of interpretation errors has not significantly improved in recent decades^1,2^. The prevalence and the impact of interpretation errors on thoracic radiographs have only seldom been investigated in veterinary medicine^4^. Conversely, this topic has been widely studied in human medicine and the most common causes of interpretation errors have been identified^5–7^. Different strategies to reduce interpretation errors have been proposed both in human^1,8^ and veterinary medicine^3^; among these is the use of computer-aided detection (CAD) tools to support the practitioner in everyday practice^6,9^.

The high performances shown by deep-learning algorithms in several radiology-related tasks have driven very active research in this field, with an increasing number of publications^10^. In particular, deep learning algorithms for the detection of specific pathologies or conditions such as pneumothorax^11^, pneumonia^12^, malignant nodules^13^ and COVID-19^14^ have been proposed. In addition, broader applications of these algorithms, such as automatic triaging^15^ and automatic labeling of chest radiographs^16^, have been investigated. Furthermore, several artificial intelligence-based products for the automatic detection of specific conditions, both on plain radiographs and computed tomographic images, have been approved by the Food and Drug Administration in the last few years, thereafter becoming commercially available.

To date, the possibilities offered by deep learning in veterinary medicine have been investigated for the classification of magnetic resonance images^17,18^ for the detection of liver degeneration from ultrasound images^19^ and for the automatic classification of corneal lesions from photographs^20^. Multi-label algorithms allow for the detection of different objects (in our case lesions) on the same image. In multi-label training each image is annotated with multiple labels according to the lesions evident on the radiograph^21^. To the best of the authors’ knowledge, both in human^11,12,22^ and in veterinary medicine^22,23^, most of the studies on applying CNNs to thoracic radiographs are focused on detecting individual pathologies or conditions, whereas studies using a multi-label approach are relatively scarce in the human medical literature^16,21,24,25^ and the scope to use multi-label algorithms on canine thoracic radiographs has not been explored yet. Therefore, the aims of this study are: (1) to develop a multi-label deep learning-based network capable of detecting some of the most common lesions found on plain radiographs of the canine thorax; (2) to test the generalization ability of the developed algorithm on an external Data Set of radiographs.

## Results

### Database

The complete database was composed of 3839 latero-lateral (LL) radiographs. Data Set 1 comprised 3063 LL images, 632 LL images were discarded due to incorrect positioning or poor image quality. Data Set 2 comprised 776 LL, 77 LL radiographs were excluded because of positioning error or poor image quality. In both data sets, “unremarkable” and “cardiomegaly” were the two most represented lesions. There was an uneven distribution of the different radiographic findings between the two data sets, with some over-represented and some under-represented in Data Set 2 when compared to Data Set 1.

**Table 1:** Number of LL radiographs showing the following included radiographic findings.

Radiographic finding	Data Set 1	Data Set 2
Unremarkable	1279	365
Cardiomegaly	583	138
Bronchial pattern	123	33
Mass	94	32
Pleural effusion	76	16
Alveolar pattern	59	41
Pneumothorax	33	12
Megaoesophagus	33	21
Pneumomediastinum	5	3
Tracheal collapse	10	2
Hernia	5	2
Fracture	5	3
Excluded	632	77

### Selection of the radiographic findings

Only a limited number of radiographs showing tracheal collapse, hernia, fracture and pneumomediastinum were available in Data Set 1 (Table 1) , and, therefore, these radiographic findings were excluded from training. Thus the radiographic findings used to train the network were: unremarkable, cardiomegaly, alveolar pattern, bronchial pattern, interstitial pattern, mass, pleural effusion, pneumothorax, megaoesophagus.

### Classification results

**Figure 1:**
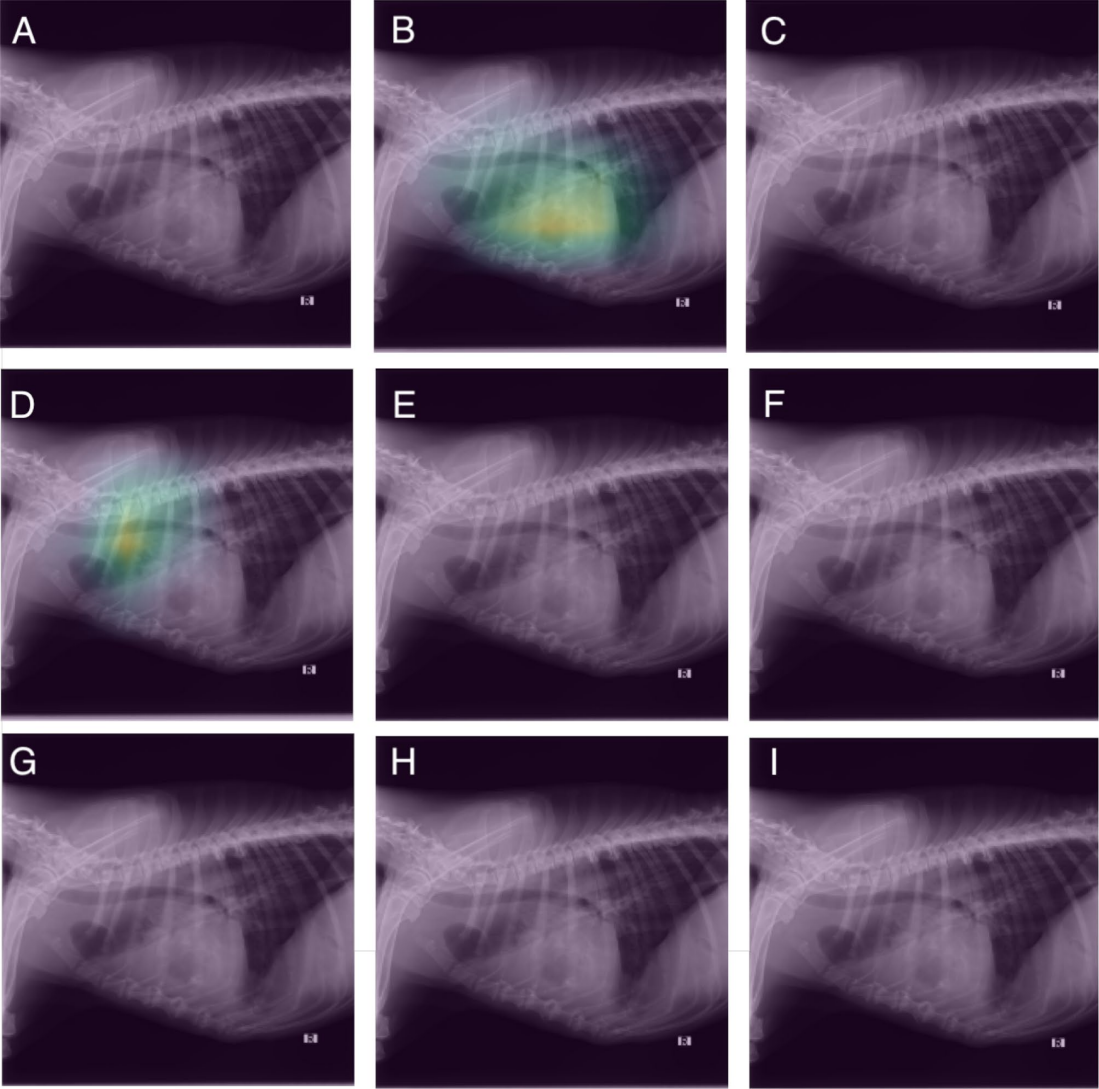
Visual assessment of the ResNet-50 classification results of a radiograph of a dog showing an alveolar pattern in the cranial lung lobe. The activations of the last layer are visualized superimposed on the radiographs. Each image corresponds to the activations for a specific radiographic finding. The alveolar pattern was correctly identified by the model (B) however the model also falsely identified the presence of a mass (E). **(A)** Original image, **(B)** alveolar pattern, **C** bronchial pattern, **(D)** cardiomegaly, **(E)** mass, **(F)** interstitial pattern, **(G)** pleural effusion, **(H)** pneumothorax, **(I)** unremarkable.

ResNet-50 had a higher classification accuracy than DenseNet-121, both on Data Set 1 and on Data Set 2, for all the considered radiographic findings except pleural effusion. Classification accuracy of the two architectures on Data Set 1 and Data Set 2 is reported in Tables 2 and 3. For some radiographic findings the classification accuracy of both ResNet-50 and DenseNet-121 was higher on Data Set 2 than on Data Set 1. In particular, both architectures showed a higher accuracy on Data Set 2 than on Data Set 1 for alveolar pattern. Furthermore, DenseNet-121 showed higher accuracy on Data Set 2 than on Data Set 1 also for bronchial pattern, cardiomegaly, megaoesophagus, unremarkable and pneumothorax. For the remaining radiographic findings, accuracy on Data Set 2 was lower than on Data Set 1. Statistically significant differences in accuracy on Data Set 2 (generalization accuracy) between ResNet-50 and DenseNet-121 were evident for: (1) alveolar pattern (Z = 3.813, P = 0.0001); (2) interstitial pattern (Z = 3.283, P = 0.0010); (3) megaeosophagus (Z = 2.257, P = 0.0240); (4) pneumothorax (Z = 3.314, P = 0.0009). No differences were evident for: cardiomegaly (Z = 0.800, P = 0.427); mass (Z = 1.580, P = 0.1142); unremarkable (Z = 0.817, P = 0.4137); pleural effusion (Z = 0.347, P = 0.7286). A graphical representation of the classification results of the model is reported in Fig. 1.

**Table 2:** Performances of ResNet-50 in Data Set 1 and Data Set 2. Parentheses show 95% CIs.

Test set	Radiographic finding	AUC	Sensitivity	Specificity	PLR	NLR
Data Set 1	Alveolar pattern	**0.87 (0.78–0.97)**	0.95 (0.64–1)	0.38 (0.31–0.45)	1.48 (1.2–1.8)	0.2(0.01–1.4)
Data Set 2	Alveolar pattern	**0.89 (0.86–0.92)**	0.95 (0.9–0.98)	0.52 (0.38–0.72)	1.99 (1.8–2.2)	0.095 (0.04–0.2)
Data Set 1	Bronchial pattern	0.78 (0.66–0.9)	0.95 (0.66–0.99)	0.092 (0.04–0.68)	1.02 (0.9–1.2)	0.78(0.1–0.54)
Data Set 2	Bronchial pattern	0.69 (0.61–0.76)	0.96 (0.86–0.99)	0.20 (0.17–0.24)	1.2 (1.1–1.3)	0.2 (0.05–0.8)
Data Set 1	Cardiomegaly	**0.92 (0.88–0.97)**	0.95 (0.86–1)	0.52 (0.43–0.6)	1.98 (1.7–2.3)	0.08 (0.02–0.3)
Data Set 2	Cardiomegaly	**0.89 (0.86–0.92)**	0.95 (0.91–0.98)	0.59 (0.54–0.63)	2.31 (2.1–2.6)	0.076 (0.03–0.2)
Data Set 1	Interstitial pattern	0.92 (0.9–0.98)	0.95 (0.52–1)	0.77 (0.71–0.83)	3.88 (2.8–5.5)	0.14 (0.02–0.9)
Data Set 2	Interstitial pattern	0.79 (0.73–0.85)	0.95 (0.87–1)	0.44 (0.4–0.48)	1.72 (1.6–1.9)	0.09 (0.02–0.3)
Data Set 1	Mass	0.77 (0.68–0.875)	0.95 (0.74–1)	0.42 (0.35–0.5)	1.64 (1.4–1.9)	0.12 (0.02–0.8)
Data Set 2	Mass	0.66 (0.55–0.77)	0.95 ( 0.85–1)	0.11 (0.09–0.14)	1.1 (1–1.2)	0.26 (0.04–1.8)
Data Set 1	Megaesophagus	0.78 (0.56–1)	0.95 (0.42–1)	0.29 (0.17–0.27)	1.10 (0.8–1.5)	0.65(0.1–4.1)
Data Set 2	Megaesophagus	0.80 (0.71–0.90)	0.95 (0.76–1)	0.31 (0.27–0.34)	1.37 (1.2–1.5)	0.16 (0.02–1.1)
Data Set 1	Pleural effusion	**0.96 (0.9–1)**	0.95 (0.64–1)	0.57 (0.49–0.63)	2.11 (1.7–2.6)	0.14 (0.02–0.9)
Data Set 2	Pleural effusion	**0.96 (0.93–0.98)**	0.95 (0.73–1)	0.81 (0.77–0.84)	4.87(4.0–5.9)	0.07 (0.01–0.5)
Data Set 1	Pneumothorax	**0.88 (0.72–0.96)**	0.95 (0.75–0.98)	0.40 (0.35–0.34)	1.56 (1.3–1.6)	0.24 (0.07–1.8)
Data Set 2	Pneumothorax	**0.84 (0.72–0.96)**	0.95 (0.64–0.96)	0.30 (0.27–0.34)	1.35 (1.2–1.5)	0.18 (0.03–1.2)
Data Set 1	Unremarkable	**0.88 (0.83–0.92)**	0.95 (0.89–0.98)	0.63 (0.54–0.73)	2.62 (2–4.4)	0.08 (0.04–0.2)
Data Set 2	Unremarkable	**0.83 (0.80–0.86)**	0.95 (0.92–0.97)	0.44 (0.38–0.5)	1.69 (1.5–1.9)	0.11(0.07–0.2)

*AUC* area under the receiver operator curve, *PLR* positive likelihood ratio, *NLR* negative likelihood ratio.

Most relevant results have been bolded.

**Table 3:** Performances of DenseNet-121 in Data Set 1 and Data Set 2. Parentheses show 95% CIs.

Test Set	Radiographic finding	AUC	Sensitivity	Specificity	PLR	NLR
Data Set 1	Alveolar pattern	0.80 (0.66–0.94)	0.95 (0.64–1)	0.33 (0.27–0.40)	1.38 (1.1–1.7)	0.23 (0.04–1.5)
Data Set 2	Alveolar pattern	0.83 (0.8–0.87)	0.95 (0.9–0.98)	0.41 (0.37–0.45)	1.61(1.5–1.7)	0.12(0.06–0.3)
Data Set 1	Bronchial pattern	0.69 (0.59–0.8)	0.95 (0.66–1)	0.44 (0.37–0.52)	1.67 (1.4–2)	0.16(0.02–1.1)
Data Set 2	Bronchial pattern	0.70 (0.63–0.77)	0.95 (0.83–1)	0.17 (0.14–0.20)	1.13 (1–1.2)	0.37 (0.1–1.1)
Data Set 1	Cardiomegaly	**0.87 (0.80–0.93)**	0.98 (0.89–1)	0.24 (0.17–0.31)	1.28 (1.2–1.4)	0.09 (0.01–0.6)
Data Set 2	Cardiomegaly	**0.98 (0.85–0.91)**	0.95 (0.87–0.96)	0.65 (0.61–0.99)	2.67 (2.4–3)	0.11(0.06–0.2)
Data Set 1	Interstitial pattern	0.78 (0.65–0.91)	0.95 (0.52–1)	0.55 (0.44–0.58)	1.82 (1.4–2.4)	0.22(0.03–1.4)
Data Set 2	Interstitial pattern	0.70 (0.64–0.77)	0.95 (0.84–1)	0.25 (0.22–0.23)	1.26(1.2–1.4)	0.23(0.08–0.7)
Data Set 1	Mass	0.64 (0.5–0.78)	0.95 (0.74–1)	0.04 (0.02–0.07)	0.98 (0.9–1.1)	1.44 (0.2–11.1)
Data Set 2	Mass	0.59 (0.49–0.7)	0.95 (0.80–1)	0.05 (0.03–0.07)	0.99(0.9–1.1)	1.27 (0.3–5.1)
Data Set 1	Megaesophagus	0.66 (0.42–0.9)	0.95 (0.36–1)	0.17 (0.1–0.22)	1 (0.7–1.4)	1 (0.2–6.1)
Data Set 2	Megaesophagus	0.69 (0.58–0.79)	0.95 (0.76–1)	0.28 (0.26–0.32)	1.32(1.2–1.5)	0.17 (0.03–1.2)
Data Set 1	Pleural effusion	0.97 (0.93–1)	0.95 (0.64–1)	0.83 (0.77–0.88)	5.51(3.9–7.8)	0.09 (0.01–0.6)
Data Set 2	Pleural effusion	0.95 (0.93–0.98)	0.95 (0.73–1)	0.89 (0.82–0.88)	6.27 (5.1–7.8)	0.06 (0.01–0.4)
Data Set 1	Pneumothorax	0.56 (0.15–0.96)	0.95 (0.73–1)	0.17 (0.07–0.63)	0.8 (0.4–1.8)	1.97 (0.4–10)
Data Set 2	Pneumothorax	0.71 (0.6–0.82)	0.95 (0.74–1)	0.22 (0.19–0.26)	1.22(1.1–1.4)	0.24 (0.04–1.6)
Data Set 1	Unremarkable	0.84 (0.79–0.9)	0.95 (0.90–0.99)	0.56 (0.46–0.66)	2.16(1.7–2.7)	0.079 (0.03–0.2)
Data Set 2	Unremarkable	0.84 (0.81–0.87)	0.95 (0.92–0.97)	0.42 (0.36–0.48)	1.63(1.5–1.8)	0.12 (0.08–0.2)

*AUC* area under the receiver operator curve, *PLR* positive likelihood ratio, *NLR* negative likelihood ratio.

Most relevant results have been bolded.

## Discussion

A new, deep learning-based, multi-label classification method for the automatic detection of several radiographic findings in canine thoracic radiographs is proposed. The high classification accuracy shown by both tested architectures on Data Set 2, for almost all the radiographic findings, suggests that multi-label CNNs can be successfully trained also in the case of relatively small-sized and highly unbalanced databases. On the other hand, the classification differences in several radiographic findings between the veterinary and the human medical literature make comparison with similar studies^21,25^ not entirely straightforward. Moreover, some of the radiographic findings that are common in humans (e.g. emphysema, fibrosis) are rarely found in dogs. Nonetheless, it is feasible to make this direct comparison between human and veterinary examples for some radiological findings, such as cardiomegaly, pleural effusion, pneumothorax, consolidation (labelled “ alveolar pattern” in this study) and unremarkable^21,25^. Interestingly, for all the above-mentioned radiographic findings, the AUC of the developed CNN was similar to or higher than that reported in similar studies on humans^21,25^ both for Data Set 1 and for Data Set 2.

Another interesting aspect of this research is related to the large variability in body size and body shape typical of the dog, which directly translates into a wide range of normality in the radiographic appearance of the canine thorax. Indeed, the dog is the only known species that has a 50-fold variability in dimensions among individuals. Therefore, it is easily understood that the radiographic appearance of the thorax of, for example, a bulldog, a dachshund, or a German shepherd, is very different in radiological terms. Despite such variability, the developed CNN was able to detect most of the radiographic findings included in the CNN with an accuracy ranging from moderate to very good. In particular, ResNet-50 displayed an AUC above 0.8 in the detection of alveolar pattern, cardiomegaly, megaoesophagus, pleural effusion, and pneumothorax. In addition it showed high accuracy in identifying normal radiographs (labelled “unremarkable”). Interestingly, in similar experiments in humans the accuracy in identifying radiologically normal images was lower^25^. Conversely, accuracy was lower than 0.8 for bronchial pattern, interstitial pattern and mass. It is the authors’ opinion that the limited generalization ability shown by ResNet-50 in the detection of bronchial and interstitial patterns might be related to the difference in image quality of the original DICOM images between Data Set 1 and Data Set 2. In fact, the radiographs acquired using the CR system had a lower image quality than those acquired through the DR system. Another possible explanation is that bronchial and interstitial patterns were not assessed on VD images. On the other hand, the low accuracy in the detection of masses could be related to the inability of the network to consider orthogonal views simultaneously. The low accuracy in detecting masses shown by ResNet-50 and DenseNet-121, both on Data Set 1 and Data Set 2, is probably related to the fact that several mass-like structures (for example nipples, degeneration of the costochondral joints in older animals, pleural mineralizations) are often present in normal radiographs. Interestingly, also in the experiments by Wang et al. 2017^24^ and Yao et al. 2018^26^ accuracy in detecting masses and nodules in humans was low (AUC below 0.8). The developed CNN had variable performances for the detection of the different lesions and, therefore, results obtained with the current version of the CNN should be confirmed with other methods (e.g.: interpretation by radiologist, computed tomography, magnetic resonance imaging) before taking clinical decisions based on those results.

ResNet-50 and DenseNet-121 are the two most commonly used pre-trained CNNs for multi-label chest X-ray image classification^21,24,26^. In this study, ResNet-50 showed a significantly higher generalization ability than DenseNet-121 in the detection of alveolar pattern, interstitial pattern, megaoesophagus, and pneumothorax, whereas no differences were evident for cardiomegaly, mass, unremarkable and pleural effusion. In previous human studies, these two network architectures demonstrated a variable accuracy in the detection of radiographic lesions ,with ResNet-50 performing better than DenseNet-121 for some lesions and vice versa^21^. Furthermore, in some studies, both ResNet-50 and DenseNet-121 were used as backbones for category-wise, residual operations, and attention-based mechanisms^21^. Incorporating the above modules within the network is reported to increase the average AUC^21^. The above modules were not included in the present study, mainly due to the limited data set size and because of the high imbalance lesion distribution.

Models trained on a specific data set do not always obtain comparable performance when tested on data sets from a different institution. Accuracy increases if the data sets acquired from multiple institutions are used for the training^27^. A limitation of this study is that both data sets were acquired at the same institution and a data set from an external veterinary clinic was not available. However, in order to keep center generalization into account, Data Set 1 and Data Set 2 (used respectively for training and testing) were acquired using two different radiograph acquisition systems. Further studies, possibly including radiographs acquired at multiple veterinary clinics, could help clarify the current generalization performances of the developed CNN. Furthermore, it is also possible that the exclusion of incorrectly positioned and exposed radiographs from both the training and the test set might have influenced the classification accuracy towards more favorable results. The possibility to automatically detect positioning or exposure abnormalities has not been explored yet.

Another limitation of the present study is that the radiographic findings included in the training set do not, of course, fully represent all the lesions types that might occur in thoracic radiographs in dogs. Furthermore, due to the limited number of available cases, radiographs showing the least represented radiographic findings (tracheal collapse, hernia, fracture, and pneumomediastinum) were not included in the training. For the above reasons, the real “in-field” generalization ability of the developed CNN has yet to be fully tested.

The developed CNN is prospectively aimed to assist veterinary clinicians, both general practitioners and radiology specialists, in their daily work. It is the authors’ opinion that the scope to use deep learning-based tools during routine clinical activity will increase productivity while decreasing the error rate. Generally speaking, veterinary facilities are smaller than human hospitals and the global number of veterinary specialists in all the disciplines is significantly lower the global number of specialist doctors. Therefore, veterinary general practitioners are required to develop expertise in several different fields of medicine, such as radiology, surgery, internal medicine, pathology, and so on. It is the authors’ opinion that, in such a scenario, veterinarians could greatly benefit from the use of deep learning-based tools to assist them in their clinical routine. Indeed, several application cases for these algorithms have been proposed and analysed in the human medical literature. For instance, the use of deep learning-based algorithms is reported to increase accuracy in the detection of pulmonary nodules by skilled radiologists^9^, or to decrease the average reporting delay in a clinical setting^15^. The possible impact CNN use in the veterinary medical field has not been evaluated yet.

## Methods

### Database creation

### Radiographic findings

All the images were reviewed by three experienced veterinary radiologists (AZ, TB and SB, with more than 20, 10 and 3, years’ experience respectively). Before interpretation, image quality was assessed and, in particular, radiograph exposure and patient positioning were evaluated. Only properly exposed images with the animal positioned correctly were included in both data sets. Radiographs of immature dogs and images with evident artefacts (double exposure, dirt on the cassette, etc.) were also excluded. When available, both LL and VD radiographs of the same patient were reviewed simultaneously. The radiographs were classified strictly based on the presence or absence of individual radiographic findings and not on the presence or absence of pathologies (e.g.: pneumonia) or conditions (e.g.: oedema) that might be characterized by the simultaneous presence of several radiographic findings. All the radiographs were labelled according to the following radiographic findings: alveolar pattern, interstitial pattern, bronchial pattern, mass, cardiomegaly, pleural effusion, pneumothorax, hernia, megaoesophagus, fracture, pneumomediastinum, tracheal collapse. If no radiographic findings were evident, the image was classified as unremarkable. The distribution (focal vs. diffused) of both alveolar and interstitial patterns was not considered. Interstitial and bronchial patterns were graded as mild, moderate, or severe. Mild bronchial and interstitial patterns were considered as normal variations in the radiographic appearance of the canine thorax and, therefore, not included in the training. If only mild bronchial and interstitial patterns were evident, the radiographs were classified as unremarkable. Cases showing both segmental and diffused megaoesophagus were classified as megaoesophagus. The presence of cardiomegaly was assessed based on the authors’ experience. In unclear cases, the vertebral heart score^28^ was calculated and then compared with the breed-specific reference intervals reported in the literature. Mediastinal and thoracic wall masses were included in the mass tag. Both diaphragmatic and abdominal wall hernias were classified as hernia. Likewise, both fractures to the ribs and to the vertebral column were classified as fracture. Fractures of the long bones were not considered. No grading score was assigned to tracheal collapse. All the images were reviewed simultaneously by the three authors and all the labels were assigned following a consensus discussion.

### Image processing and deep learning

The deep-learning analysis was performed on a dedicated workstation (Linux operating system, Ubuntu 18.04, Canonical) equipped with four graphic processing units (Tesla V100; NVIDIA), a 2.2 GHz processor (Intel Xeon E5-2698 v4; Intel) and 256 GB random-access memory. Before feeding to the CNN the images were downsampled to 224x224 pixels. The images were not cropped during the test phase, neither lossy compressed or converted to JPEG. Instead, the lossless MHA format was used. Radiograph classification was performed using convolutional neural networks (CNN), a special class of deep-learning algorithms specifically designed to work with images, and this classification was performed using two different CNN architectures: (1) DenseNet-121^29^, (2) ResNet-50^30^. The tested CNN architectures were pre-trained on a large-scale data set of everyday images called ImageNet and then fine-tuned. Different radiographic findings are usually evident on the same radiograph, often as a result of a single condition or pathology, and, therefore, a multi-label approach was used. Binary cross-entropy was used as the objective function. The same training parameters were used for all the networks. Training was performed until convergence using the Adam optimizer and a learning-rate scheduler with exponential decay. The weights from the epoch with the lowest loss on the validation set were chosen and further used for testing. The training set was augmented by random horizontal/vertical flips, cropping, affine warping, and linear contrast changes. All the images were normalized to the 0-1 range, where 0 denotes the background. The split ratio for training, validation, and test set (for Data Set 1) was 8:1:1 respectively.The training scheme was not directly optimizing any of the evaluation metric, e.g. AUC, sensitivity, or specificity. No information from Data Set 2 was used during the training.

### Statistical analysis

We assessed individual architectures, both on Data Set 1 and Data Set 2, with the area under the receiver operating characteristic curve (AUC) using a commercially available statistical software (MedCalc). Sensitivity was calculated as: true positive /(true positive $+$ false negative), specificity as: true negative/ (false positive $+$ true negative), positive likelihood ratio (PLR) as: sensitivity / (1 − specificity) and negative likelihood ratio (NLR) as: (1 − sensitivity)/specificity. The performances of the two architectures were compared, on the Data Set 2 only, with the DeLong test. The differences in the AUCs of the considered tests, as a result of the DeLong test, are expressed as Z score. All p-values were assessed at an alpha of 0.05.

## Conclusions

A multi-label CNN-based network for the automatic classification of canine LL radiographs was developed and tested. The developed network had a variable accuracy in the detection of radiographic findings in an external test set. Further studies, hopefully including a larger number of radiographs acquired in several different veterinary institutions, could allow the development of a network with a broader generalization ability. Furthermore, a larger database could allow testing the network also on VD images. CNN-based tools could, prospectively, assist the veterinarian in his everyday work allowing for a higher quality veterinary care. Nonetheless, for a successful application of these tools in the clinical workflow, the advantages and the pitfalls of such tool must be clearly known by the operator.

## Data Availability

The data sets generated during and/or analysed during the current study are not publicly available because they are property of the Veterinary Teaching Hospital of the University of Padua but are available from the corresponding author on reasonable request.
